# *ENPP1* K121Q (rs1044498 C > A) genetic polymorphism confers a high risk of susceptibility to coronary heart disease: A PRISMA-compliant article

**DOI:** 10.1097/MD.0000000000011236

**Published:** 2018-07-06

**Authors:** Jia-Yin Di, Meng-Lu Dai, Zong-Xin Zhang

**Affiliations:** a Clinical Laboratory, Department of Outpatient, Huzhou University; b Department of Clinical Laboratory, Huzhou Central Hospital, Huzhou, P.R. China.

**Keywords:** coronary heart disease, *ENPP1*, genetic polymorphism, K121Q, meta-analysis, rs1044498 C > A

## Abstract

**Background:**

Previous studies suggested an association between K121Q (rs1044498 C > A) in ecto-nucleotide pyrophosphatase phosphodiesterase 1 (ENPP1) gene and the risk of coronary heart disease (CHD), but the results have been inconsistent. In this study, we performed a meta-analysis of several trials to systematically summarize their potential association.

**Methods:**

Relevant articles were identified by searching electronic databases for studies published prior to March 2018. We carefully reviewed published studies on ENPP1 genetic polymorphism in relation to CHD susceptibility. The data extracted from selected high-quality studies were analyzed using STATA statistical software (StataCorp LP, College Station, TX, USA).

**Results:**

Nine eligible studies which contained a combined total of 1547 CHD cases and 2213 healthy controls were chosen in the present meta-analysis. Our results indicated that K121Q strongly correlated with increased risk of CHD. The subgroup analysis on race, sample source, disease type, sex, age, and genotype showed that in Caucasians, K121Q strongly correlated with increased risk of CHD, but no difference was found in Chinese. Both single factor and multiple factor regression showed that race, sample origin, disease type, sex, age, and genotype were not the source of heterogeneity.

**Conclusions:**

Our meta-analysis revealed that the K121Q (rs1044498 C > A) in the ENPP1 gene is a risk factor for CHD.

## 1 Introduction

Coronary heart disease (CHD), also variously known as atherosclerotic heart disease, coronary artery disease, or ischemic heart disease, is the most common type of heart disease and the leading cause of death worldwide.^[1–4]^ CHD is associated with plaque formation along the inner walls of coronary arteries, leading to narrowing of the arteries and limitation of blood flow to the heart.^[5]^ It is estimated that about 620,000 Americans have a new coronary attack annually and approximately 295,000 have a recurrent attack each year.^[6]^ In Europe, CHD is responsible for approximately 4 million deaths annually. Although the exact mechanisms involved in CHD are not completely understood, the increased incidence of CHD is attributed to a variety of environmental and genetic factors.^[7]^ Other important extrinsic risk factors, such as high blood cholesterol/pressure, smoking, diabetes, physical inactivity, obesity, poor diet, age, and medical history, are also indispensable factor for CHD.^[6,8]^ In the past 2 decades, various genetic factors have been verified as significant contributors to the development of CHD.^[9]^ Recent findings support the view that ectonucleotide pyrophosphatase phosphodiesterase 1 (*ENPP1*) gene is associated with increased susceptibility to CHD in humans, and the mutations in *ENPP1* gene are related to insulin resistance as well as idiopathic infantile arterial calcification.^[10,11]^

ENPP1 is a type II transmembrane glycoprotein with extracellular pyrophosphatase and phosphodiesterase activities.^[12]^ The human *ENPP1* gene is 80 kb in length and is located on chromosome 6q22-q23, consisting of 25 exons and 24 introns, and encoding a 925-amino acid protein with a molecular weight of 104.9 kDa.^[13]^*ENPP1* is expressed in multiple tissues, including muscle, capillary endothelium in the brain, fat, salivary duct epithelium, liver, adipose tissue, pancreas, chondrocytes, and kidneys.^[14]^ Elevated *ENPP1* expression is found in adipose tissue, cultured skin fibroblasts, and skeletal muscle of insulin-resistant individuals, suggesting that over-expression of *ENPP1* may be an early marker of insulin resistance in humans.^[13,15]^ Insulin resistance is a critical risk factor in the development of type 2 diabetes mellitus, and is also related with obesity, dyslipidemia, hypertension, and coronary atherosclerosis.^[16]^ The underlying mechanisms appear to be related to ENPP1-mediated inhibition of insulin receptor tyrosine kinase activity, and overexpression of ENPP1 decreases the activity and cellular signaling of insulin receptor tyrosine kinase, inducing insulin resistance and contributing to the development of type 2 diabetes mellitus.^[17]^ Further, elevated *ENPP1* expression promotes vascular inflammatory responses, inflammatory cytokine secretion, and left ventricular mass through stimulation of insulin like growth factor-1 receptors, which are abundantly expressed in the myocardium and then subsequently increase cardiovascular risk.^[18]^ Genetic researches also support that *ENPP1* is a vital biomarker of insulin resistance, and *ENPP1* genetic polymorphism may be correlated with type 2 diabetes mellitus, nephrovascular complications, and cardiovascular disease.^[19,20]^ Recently, several studies have suggested the possibility that *ENPP1* genetic polymorphisms are correlated with significantly elevated risk of CHD,^[21,22]^ however, the results are inconsistent with the results of other studies.^[23,24]^ In this study, we employ a meta-analysis based approach to summarize the relationship between CAD and *ENPP1* K121Q (rs1044498 C > A), as a prelude to the development of novel strategies for prevention and treatment of CHD.

## 2 Materials and methods

### 2.1 Literature search

The databases such as Web of Science, CINAHL, PubMed, Cochrane Library, EMBASE, and Chinese Biomedical (CBM) were searched to identify case-control studies, which were published prior to March 2018. The combination of keywords and MeSH terms used for search strategy were: (“genetic polymorphism” or “SNP” or “variation” or “single nucleotide polymorphism” or “polymorphism” or “mutation” or “variant”) and (“ectonucleotide pyrophosphatase phosphodiesterase 1” or “ectonucleotide pyrophosphatase phosphodiesterase 1” or “plasma cell membrane glycoprotein PC-1” or “nucleotide pyrophosphatase-alkaline phosphodiesterase I” or “glycoprotein PC-1” or “ENPP1” or “plasma-cell membrane glycoprotein 1” or “PC-1” or “PC-1 glycoprotein”) and (“Myocardial Infarction” or “Coronary Artery Disease ” or “CAD” or “MI” or “myocardial infarct” or “myocardiac infarction” or “myocardium infarction” or “cardiac infarction” or “myocardia infarction” or “infarction myocardium” or “myocardial infarcted” or “heart infarction” or “heart infarction” or “Myocardial Infarction” or “acute myocardial infarction” or “Coronary Heart Disease” or “CHD” or “AMI”). Additionally, a manual cross-reference search of the references of the relevant articles was performed to identify studies beside the computerized search.

### 2.2 Inclusion and exclusion criteria

All selected studies in this meta-analysis met the following inclusion criteria: studies reporting CHD and *ENPP1* K121Q (rs1044498 C > A); studies were case-control design; all patients were confirmed by the diagnostic criteria of CHD; studies supplying sufficient information on *ENPP1* K121Q. The following studies were excluded: letters, reviews, case reports, conference abstracts, editorials, or expert opinions; studies in languages other than Chinese or English; studies on polymorphisms of *ENPP1* not relevant to this study. In addition, we chose the most recent paper when multiple articles with same data were reported by the same author.

### 2.3 Data extraction and methodological assessment

The following data were extracted: first author, publication year, country, ethnicity, number of cases and controls, sex, age, genotype method, gene, and Newcastle-Ottawa Scale (NOS) score. NOS criteria was used by 2 coauthors to evaluate the methodological quality of the included studies.^[25]^ Two researchers independently extract the document data and make the NOS quality evaluation. If the data extraction process or NOS quality evaluation is controversial, a number of researchers discussed and solve the controversial problems.

### 2.4 Statistical analysis

The unadjusted relative risks (RRs) and its corresponding 95% confidence interval (CI) was adopted to estimate the strength of the relation between the *ENPP1* K121Q and CHD based on the genotype frequencies in the 2 groups. Subgroup analysis by country, source of controls, disease, and genotype method was performed. Fixed-effect or random-effect model were applied to calculate pooled RRs. Statistical significance of pooled RRs was determined by *Z* test. The possibility of heterogeneity was evaluated using the Cochran's *Q*-statistic and *I*^*2*^ tests.^[26]^*P* value <.05 or *I*^*2*^ > 50% meant obviously heterogeneity, and then a random-effect model was employed, otherwise a fixed-effect model was employed. Sensitivity analyses were performed to investigate potential source of the heterogeneity. Publication bias was also studied by visual inspection of funnel plots as well as Egger test.^[27]^

## 3 Results

### 3.1 The characteristics of included studies

**Figure 1:**
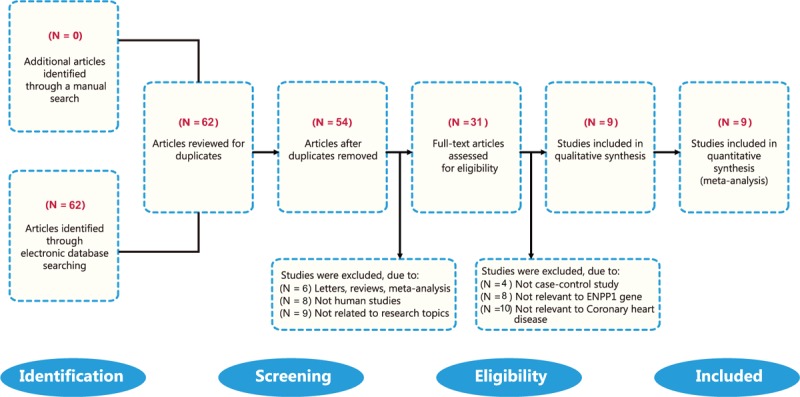
Flow chart of literature search and study selection. Six case-control studies were included in this meta-analysis.

A total of 62 relevant studies were identified in the initial search. Based on the inclusion and criteria, 53 articles were removed and 9 case-control studies were finally enrolled into this meta-analysis.^[18,20–22,24,28–31]^ The details of the study selection process are presented in Fig. 1. The 9 selected studies contained a combined total of 1547 CHD cases and 2213 healthy controls. All patients met the diagnostic criteria of CHD confirmed by pathological examination of the surgical specimen. Overall, 3 studies involved Asian populations and 6 studies in Caucasians. The source of sample in all the selected studies was blood. Three methods were used for genotyping including mutagenically separated polymerase chain reaction (MS-PCR), TaqMan, and PCR-restriction fragment length polymorphism (PCR-RFLP). Hardy-Weinberg Equilibrium (HWE) tests were performed in all included studies. All studies were evaluated as NOS scores ≥5. The characteristics as well as methodological quality of the enrolled studies are demonstrated in Table 1.

**Table 1:**
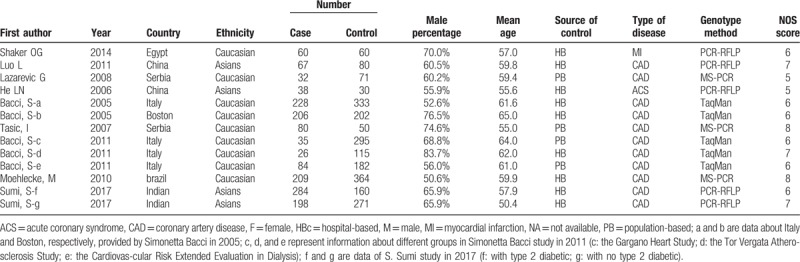
Main characteristics and methodological quality of case-control studies.

### 3.2 Association between the ENPP1 K121Q (rs1044498 C > A) and CHD risk

**Figure 2:**
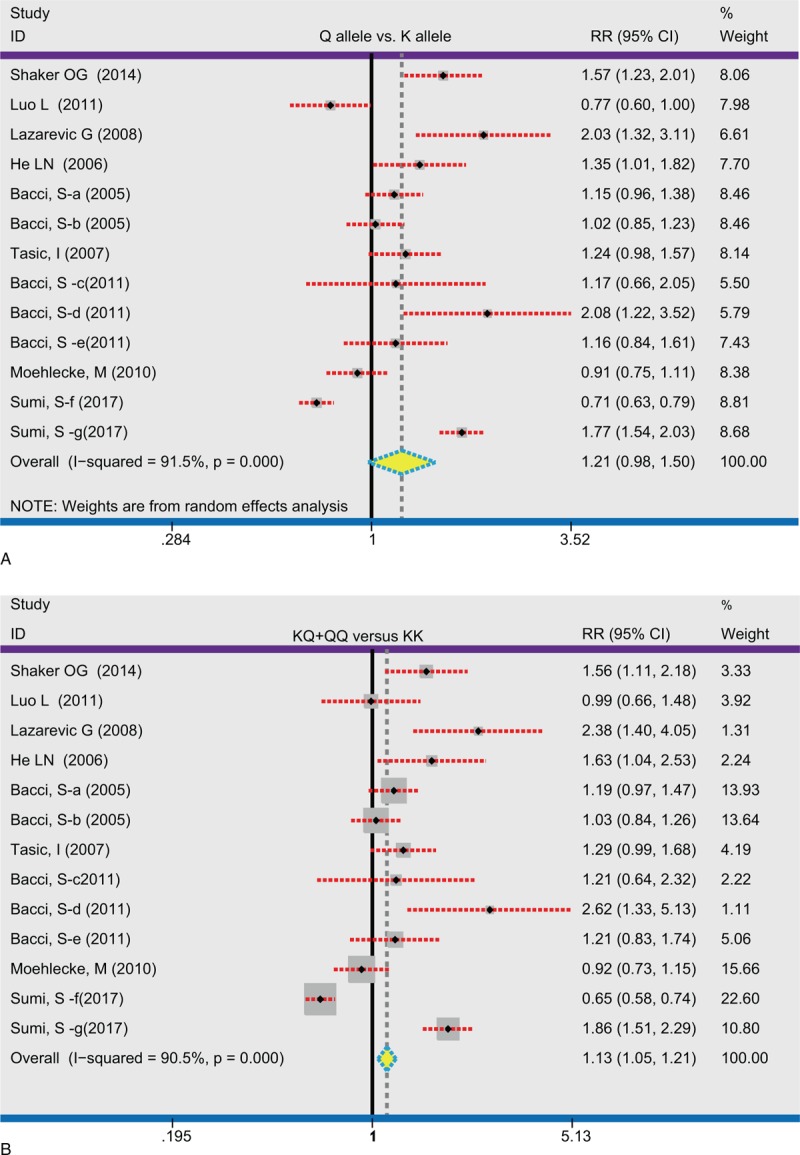
Forest plots for the relationships between *ENPP1* K121Q and coronary heart disease risk under the allele and dominant model. A: allele model, B: dominant model.

A summary of the study findings of the relation between the *ENPP1* K121Q and CHD risk is provided in Table 2. The meta-analysis results revealed that *ENPP1* K121Q showed a significant correlation with the CHD risk (dominant model: RR = 1.13, 95%CI = 1.05–1.21, *P* = .001) (Fig. 2B).

**Table 2:**
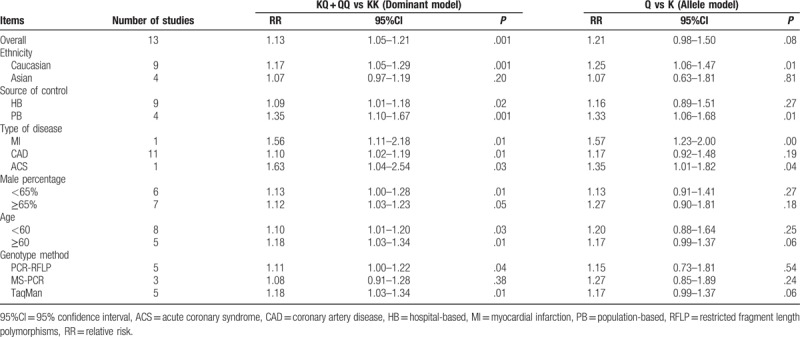
Meta-analysis of the relationships between *ENPP1* genetic polymorphism and the coronary heart disease.

**Figure 3:**
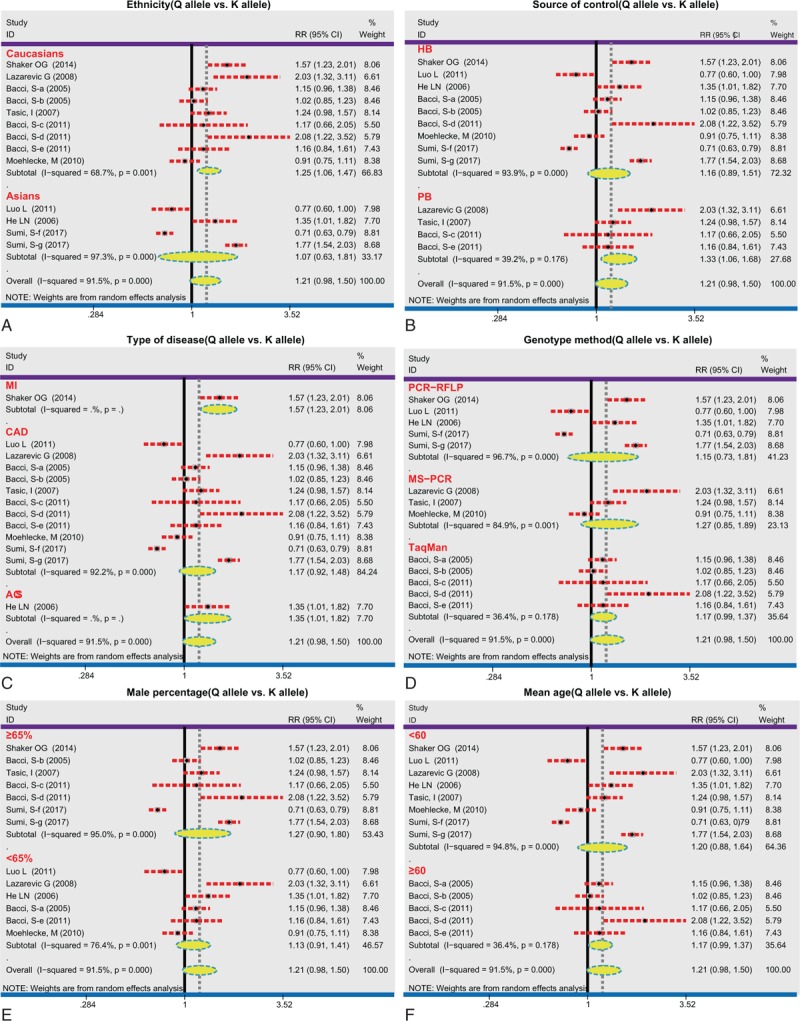
Subgroup analyses by country, disease, genotyping method, and sources of control of the relationships between *ENPP1* K121Q and coronary heart disease risk under the allele model. A: ethnicity, B: source of control, C: type of disease, D: genotype method, E: male percentage, F: mean age. 95%CI = 95% confidence interval; ACS = acute coronary syndrome; CAD = coronary artery disease; HB = hospital-based; MI = myocardial infarction; PB = population-based; RFLP = restricted fragment length polymorphisms; RR = relative risk.

**Figure 4:**
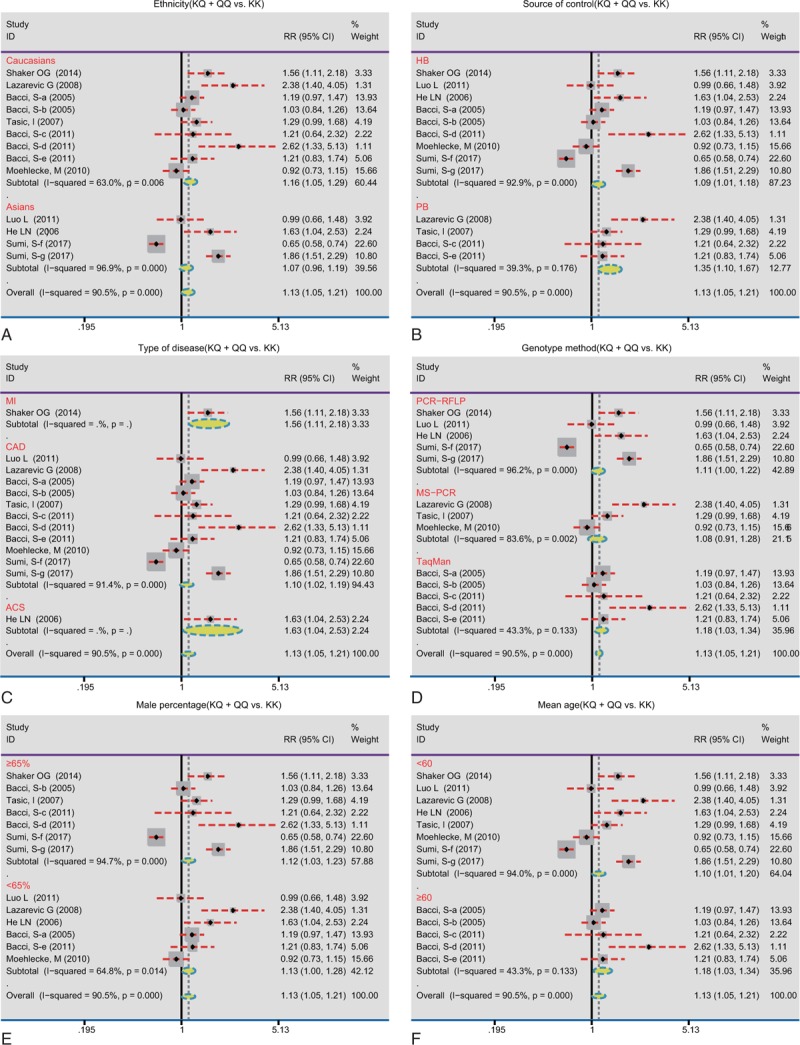
Subgroup analyses by country, disease, genotyping method, and sources of control of the relationships between *ENPP1* K121Q and coronary heart disease risk under the dominant model. A: ethnicity, B: source of control, C: type of disease, D: genotype method, E: male percentage, F: mean age. 95%CI = 95% confidence interval; ACS = acute coronary syndrome; CAD = coronary artery disease; HB = hospital-based; MI = myocardial infarction; PB = population-based; RFLP = restricted fragment length polymorphisms; RR = relative risk.

We also conducted the following stratified analyses for *ENPP1* K121Q (Table 2). In the allele and dominant model, we found a significant correlation of the *ENPP1* K121Q with CHD risk in Caucasian (allele model: RR = 1.25, 95%CI = 1.06–1.47, *P* = .01; dominant model: RR = 1.17, 95%CI = 1.05–1.29, *P* = .001; respectively), while no such association was detected among Asian (*P* > .05) (Figs. 3 and 4Figs. 3A and 4A). We found an association between the *ENPP1* K121Q and CHD risk among the population-based subgroup and hospital-based subgroups (all *P* < .05) (Figs. 3B and 4B). Additionally, we detected that *ENPP1* K121Q was related to the risk of MI, CAD, and acute coronary syndrome (ACS) in the dominant model (all *P* < .05) (Figs. 3C and 4C). With regard to the genotype analysis methods, *ENPP1* K121Q showed a significant association with CHD risk using PCR-RFLP and TaqMan (PCR-RFLP: RR = 1.11, 95%CI = 1.00–1.22, *P* = .04; TaqMan: RR = 1.11, 95%CI = 1.00–1.22, *P* = .04), but not with MS-PCR (all *P* > .05) (Fig. 4F). With regard to the male percentage, *ENPP1* K121Q showed a significant correlation with CHD risk (<65%: RR = 1.13, 95%CI = 1.00–1.28, *P* = .01; ≥65%: RR = 1.12, 95%CI = 1.03–1.23, *P* = .05) (Fig. 4E). With regard to the age, *ENPP1* K121Q showed a significant association with CHD risk (<60: RR = 1.10, 95%CI = 1.01–1.20, *P* = .03; ≥60: RR = 1.18, 95%CI = 1.03–1.34, *P* = .01) (Fig. 4F). We did single factor regression and multiple regression on the number of years of publication, race, sample origin, disease, genotyping, male ratio and average age, and found that these were not heterogeneous factors in the study (Table 3).

**Table 3:**
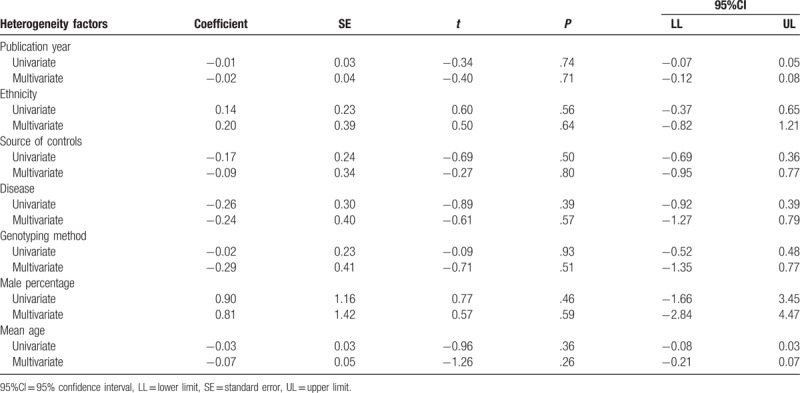
Univariate and multivariate meta-regression analyses of potential source of heterogeneity.

### 3.3 Sensitivity analysis and publication bias

**Figure 5:**
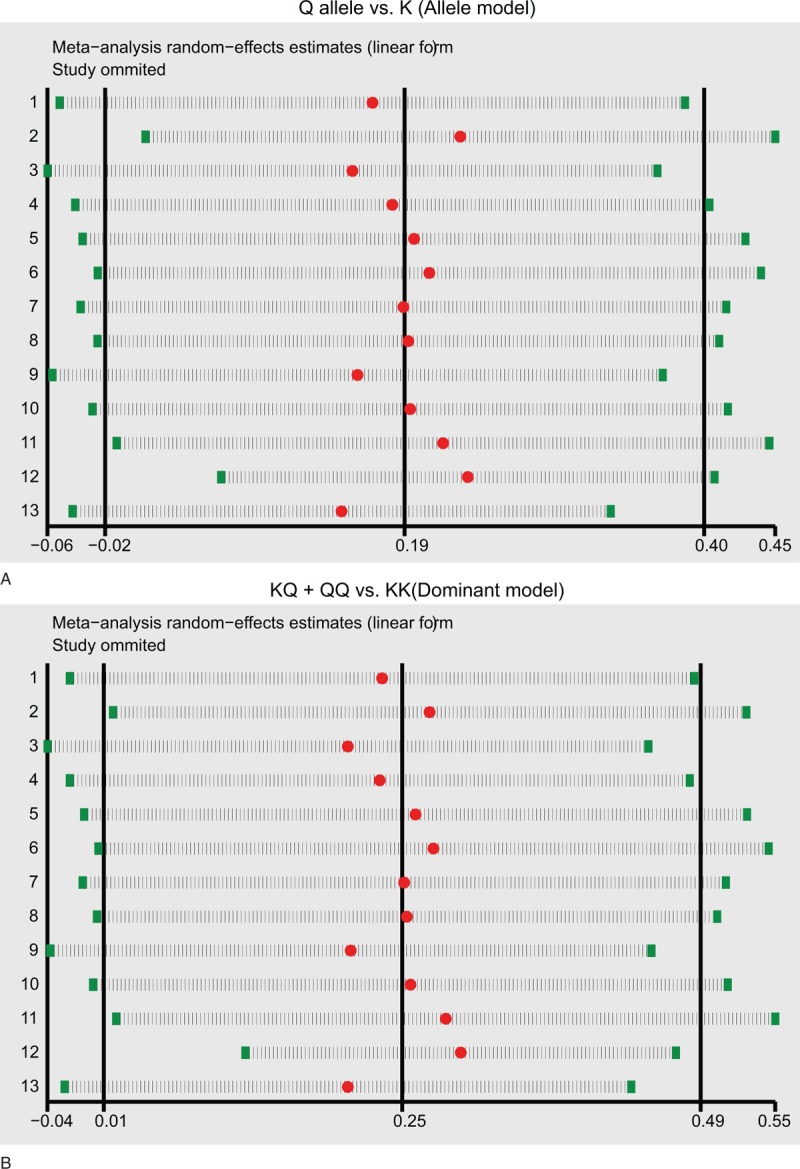
Sensitivity analysis of the summary odds ratio coefficients on the relationships between ENPP1 K121Q and coronary heart disease risk under the allele and dominant models. ENPP1 = ecto-nucleotide pyrophosphatase phosphodiesterase 1.

**Figure 6:**
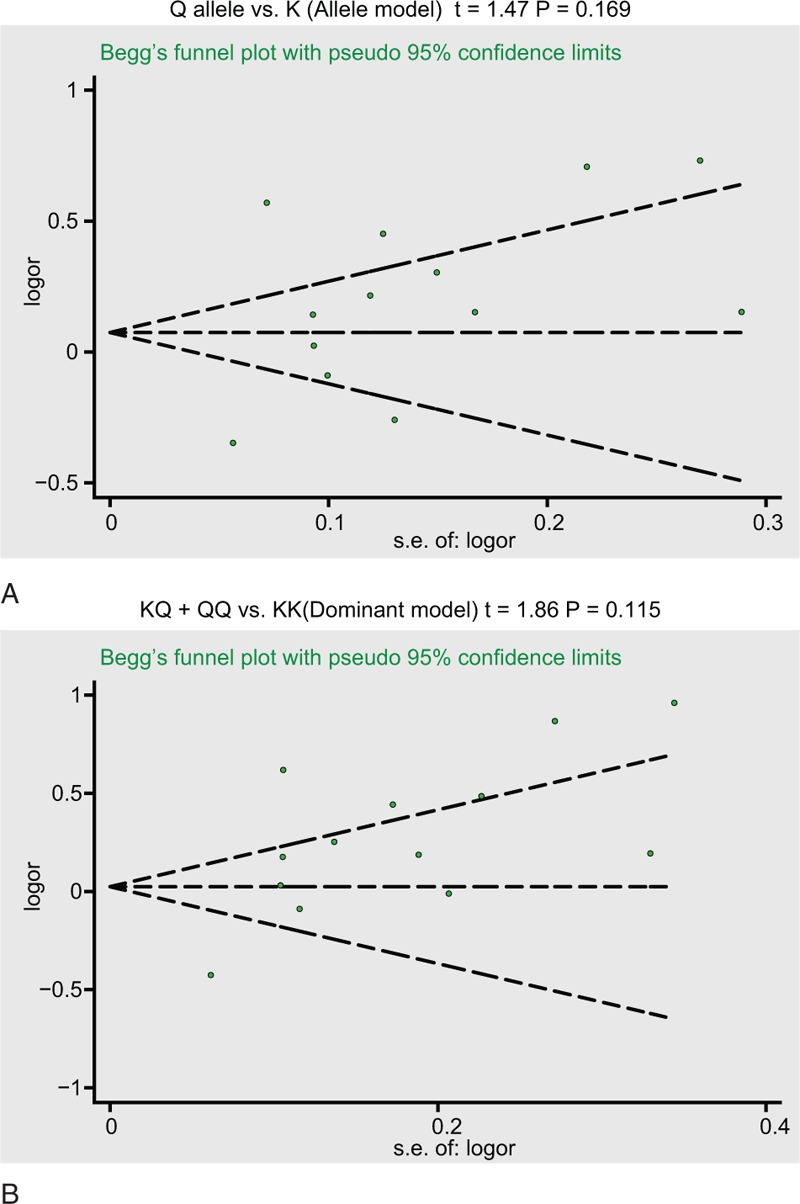
Funnel plot of publication biases on the relationships between ENPP1 K121Q and coronary heart disease risk under the allele and dominant models. ENPP1 = ecto-nucleotide pyrophosphatase phosphodiesterase 1.

Sensitivity analyses evaluated the effect of each article on the pooled ORs by excluding individual studies. The analysis results indicated no individual article significantly influenced the pooled ORs of *ENPP1* K121Q (Fig. 5). Funnel plot as well as Egger test were applied to estimate publication bias of the selected studies. The funnel plots of *ENPP1* K121Q m revealed no presence of obvious asymmetry. Moreover, Egger test failed to test publication bias (all *P* > .05) (Fig. 6).

## 4 Discussion

The relationship between *ENPP1* gene and CHD risk has been investigated previously in multiple studies, while at the moment there is a heated debate, but no consensus of that issue. We performed this meta-analysis to provide a comprehensive evaluation of the relationship between *ENPP1* K121Q and the risk of CHD by combining the data from previous studies and deriving reliable conclusions based on our statistical analysis. In the present meta-analysis, our results demonstrated that *ENPP1* K121Q was responsible for a significantly increased risk of CHD, suggesting that the genetic polymorphism of *ENPP1* could be helpful in predicting the pathogenesis of CHD. It was established that *ENPP1* gene inhibited insulin receptor tyrosine kinase activity, and promoted insulin resistance.^[32]^ Insulin resistance was a vital factor in the etiology of cardiovascular diseases.^[33]^ Insulin resistance was widely accepted as largely contributed by genetic factors, and from this study, we also regarded *ENPP1* genetic variant as one of the genetic factors, which might contribute to impaired sensitivity to insulin and then resulted in predisposition of individuals to CHD.^[22]^ The current meta-analysis mainly described the *ENPP1* K121Q, which resulted in amino acid substitution to glutamine from lysine at codon 121. The *ENPP1* Q variant had a stronger binding affinity to insulin receptor as well as decreased its auto-phosphorylation, compared with the K variant. It could be speculated that the *ENPP1* K121 Q variant might increase cardiovascular risk for causing systemic insulin resistance as well as proatherogenic phenotypes.^[34]^ On the other hand, a direct mechanism related to CHD could also be involved due to the influence of the polymorphism on insulin-dependent endothelial function.^[18]^ From the above discussion, stronger binging of the K121Q variant to the insulin receptor at the cell membrane inhibited insulin signaling.^[35]^ In human endothelial cells harboring the K121Q variant, the consequences were serious because suppression of insulin receptor signaling potentially impacted synthesis and release of nitric oxide, and a powerful vasodilator whose decreased levels contributed to the development of atherosclerosis.^[36]^ Therefore, we hypothesized that the K121Q variants in the *ENPP1* gene significantly might increase the susceptibility to CHD through its systemic effects and endothelial-specific effects, leading to insulin resistance and contributing to rapid progression of CHD. Consistent with our results, Shaker and Ismail^[22]^ showed in 60 unrelated patients suffering from their first MI and 60 unrelated controls, that the K121Q variant conferred a higher risk of early development of insulin resistance and patients showed significantly faster progression of acute myocardial infarction compared with the 121K allele. The present study clearly revealed that the occurrence of the *ENPP1* K121Q polymorphism was significantly higher in CHD patients and the *ENPP1* K121Q variant could be a clinically useful biomarker for population-based screening to identify high-risk individuals for their susceptibility to major cardiovascular events. Subgroup analysis by country showed a significant relation of K121Q polymorphism with CHD risk in Caucasian, but not in Asian. The results can be explained that individuals in different countries may have different genetic backgrounds and life-styles.

Limitations of the present study should be considered. Publication bias may result from the fact that unpublished data, as well as papers published in languages other than English and Chinese, were not included. Further, our meta-analysis division criteria of ethnic groups into “Caucasian,” or “Asian” may bias the results, without the detailed knowledge of the patients. Another limitation in our meta-analysis is the small sample size. Despite these limitations, we quantified and analyzed previous inconsistent results from previous studies in our meta-analysis. Besides, the number of years of publication, race, sample origin, disease, genotyping, male ratio, and average age were not heterogeneous factors in this study, which indicates a more credible conclusion of the relationship between *ENPP1* genetic polymorphism and CHD in our results.

In conclusion, our results revealed that *ENPP1* K121Q confers a high risk of susceptibility to CHD and may be useful in early identification of at-risk CHD population, especially in Caucasian. These results indicated that *ENPP1* genetic variation might be crucial in the occurrence of CHD and the genetic variant might be helpful in understanding the basic biology leading to insulin resistance. However, future studies including larger sample size and different ethnicities are also needed to confirm our findings and begin to develop therapeutic intervention strategies focused on *ENPP1* K121Q.

## Acknowledgments

The authors would like to give our appreciation to the reviewers for their helpful comments.

## Author contributions

**Conceptualization:** Jia-Yin Di.

**Data curation:** Jia-Yin Di.

**Formal analysis:** Jia-Yin Di, Meng-Lu Dai.

**Funding acquisition:** Meng-Lu Dai.

**Investigation:** Meng-Lu Dai, Zong-Xin Zhang.

**Methodology:** Zong-Xin Zhang.

**Project administration:** Zong-Xin Zhang.
